# Role of the Purkinje-Muscle Junction on the Ventricular Repolarization Heterogeneity in the Healthy and Ischemic Ovine Ventricular Myocardium

**DOI:** 10.3389/fphys.2018.00718

**Published:** 2018-06-14

**Authors:** Marine E. Martinez, Richard D. Walton, Jason D. Bayer, Michel Haïssaguerre, Edward J. Vigmond, Mélèze Hocini, Olivier Bernus

**Affiliations:** ^1^Centre de Recherche Cardio-Thoracique de Bordeaux, Université de Bordeaux, Bordeaux, France; ^2^INSERM U1045, Centre de Recherche Cardio-Thoracique de Bordeaux, Bordeaux, France; ^3^IHU Liryc, Electrophysiology and Heart Modeling Institute, Fondation Bordeaux Université, Bordeaux, France; ^4^UMR5251, Centre National De La Recherche Scientifique, Institut de Mathématiques de Bordeaux, Bordeaux, France; ^5^Centre Hospitalier Universitaire, Bordeaux University Hospital, Hopital Cardiologique du Haut Lévèque, Bordeaux, France

**Keywords:** transmural heterogeneity, activation sequence, Purkinje-muscle junction, repolarization, optical mapping

## Abstract

Alteration of action potential duration (APD) heterogeneity contributes to arrhythmogenesis. Purkinje-muscle junctions (PMJs) present differential electrophysiological properties including longer APD. The goal of this study was to determine if Purkinje-related or myocardial focal activation modulates ventricular repolarization differentially in healthy and ischemic myocardium. Simultaneous epicardial (EPI) and endocardial (ENDO) optical mapping was performed on sheep left ventricular (LV) wedges with intact free-running Purkinje network (*N* = 7). Preparations were paced on either ENDO or EPI surfaces, or the free-running Purkinje fibers (PFs), mimicking normal activation. EPI and ENDO APDs were assessed for each pacing configuration, before and after (7 min) of the onset of no-flow ischemia. Experiments were supported by simulations. In control conditions, maximal APD was found at endocardial PMJ sites. We observed a significant transmural APD gradient for PF pacing with PMJ APD = 347 ± 41 ms and EPI APD = 273 ± 36 ms (*p* < 0.001). A similar transmural gradient was observed when pacing ENDO (49 ± 31 ms; *p* = 0.005). However, the gradient was reduced when pacing EPI (37 ± 20 ms; *p* = 0.005). Global dispersion of repolarization was the most pronounced for EPI pacing. In ischemia, both ENDO and EPI APD were reduced (*p* = 0.005) and the transmural APD gradient (109 ± 55 ms) was increased when pacing ENDO compared to control condition or when pacing EPI (*p* < 0.05). APD maxima remained localized at functional PMJs during ischemia. Local repolarization dispersion was significantly higher at the PMJ than at other sites. The results were consistent with simulations. We found that the activation sequence modulates repolarization heterogeneity in the ischemic sheep LV. PMJs remain active following ischemia and exert significant influence on local repolarization patterns.

## Introduction

Action potential duration heterogeneity is thought to play an important role in coordinating repolarization of the ventricles. In the healthy heart, the intrinsic APD of endocardial myocytes has been found to be longer than for epicardial cells, in most mammalian species investigated (Nerbonne and Kass, 2005). This gradient can be explained by a differential expression of ion channels between these cells. Cardiovascular diseases, such as acute ischemia, are known to lead to abnormal repolarization heterogeneity in ventricles due to acute or longer term electrical remodeling at the level of ventricular myocytes, decreasing cellular coupling, reducing conduction velocities, and increasing APD heterogeneity (Cascio et al., 1995; Shaw and Rudy, 1997). An additional source of heterogeneity in the intact ventricular myocardium is PFs, which present differential electrophysiological properties including longer APD (Myerburg et al., 1970; Dun and Boyden, 2008). APD prolongation has been observed at the PMJ and attributed to electrotonic loading effects (Walton et al., 2014).

Action potential duration gradients have been shown to be further modulated by the activation sequence in small mammals (Myles et al., 2010; Walton et al., 2013), where a strong relationship between APD and AT has been observed. This suggests that in intact tissue, electrotonic modulation of APD dominates the effects of intrinsic differences in cellular repolarization characteristics (Myles et al., 2010). Those findings remain to be confirmed in large mammals during normal or ectopic activation.

Heterogeneity of repolarization is associated with the vulnerability to arrhythmias (Surawicz, 1997). Furthermore, the degree of heterogeneity often undergoes significant changes in disease, such as myocardial infarction and ischemic heart disease, known to be pro-arrhythmic. Arrhythmias during acute myocardial ischemia occur in two phases, the 1A phase between 2 and 10 min following coronary artery occlusion and the 1B phase between 18 and 30 min. The major cause for the 1A arrhythmias is the depression of excitability and conduction velocity due to the decrease in resting potential and the marked heterogeneity in refractoriness (Janse et al., 2003). Arrhythmias in phase 1B are associated with intercellular electrical uncoupling, mediated by decreased conductance of gap junction channels (De Groot and Coronel, 2004).

Although the resistance of PF toward ischemic insults due to a high metabolic reserve is well known (Tanaka et al., 2005; Ono et al., 2009), as well as its potential to trigger arrhythmias (Haissaguerre et al., 2002a), the role of repolarization heterogeneity at the PMJ in providing an arrhythmogenic substrate during ischemia remains to be elucidated.

We hypothesized that the activation sequence plays an important role in determining the dispersion of repolarization in healthy large mammals and that local repolarization heterogeneity at the PMJ is augmented during ischemia due to the increased APD gradient between ischemia-resistant PF and ischemic myocardium. We investigated this hypothesis by optical mapping experiments from left ventricular (LV) wedge preparations of sheep with activation originated from the endocardium, epicardium, or free-running PF, mimicking normal anterograde activation. We repeated these measurements after the onset of acute no-flow ischemia. Finally, we simulated optical mapping experiments for direct comparison with experimental recordings.

## Materials and Methods

### Experimental Preparation

This study was carried out in accordance with the recommendations of the Directive 2010/63/EU of the European Parliament on the protection of animals used for scientific purposes and approved by the local ethical committee of Bordeaux CEEA50. Hearts were obtained from female adult sheep (*N* = 7; 45–62 kg). Sheep were pre-medicated with ketamine (20 mg/kg) and acepromazine (Calmivet, 1 mL/50 kg). Anesthesia was induced with intraveneous injection of sodium pentobarbital (10 mg/kg) and maintained under isofluorane, 2%, in 100% O_2_. Sheep were euthanized by sodium pentobarbital (40 mL, from 50 mg/mL of stock) and the heart rapidly excised, cannulated by the aorta, and rinsed with cold cardioplegic solution, containing (mM): NaCl, 110; CaCl_2_, 1.2; KCl, 16; MgCL_2_, 16; NaHCO_3_, 10; and glucose, 9.01 at 4°C. The LV wall was dissected and cannulated by the left anterior descending artery. LVs were mounted on to a frame and were submersed (thus mimicking intracardiac blood pool and pericardial fluid) and perfused (20 mL/min) with a warm (37°C) saline solution containing (mM): NaCl, 130; NaHCO_3_, 24; NH_2_PO_4_, 1.2; MgCl_2_, 1; glucose, 5.6; KCl, 4; CaCl_2_, 1.8; gassed with 95% O_2_/5% CO_2_ at 37°C (pH 7.4); and supplemented with blebbistatin (10 μM), to inhibit contraction.

### Optical Mapping Setup and Protocol

Ventricles were stained with the potentiometric dye di-4-ANBDQBS (10 μM) which was excited by illumination of the endocardial and epicardial surfaces using monochromatic LEDs at 660 nm (Cairn Research Ltd., Kent, United Kingdom). Two Micam Ultima CMOS cameras (SciMedia USA Ltd., Costa Mesa, CA, United States) with a 100 × 100 pixels resolution were used to obtain simultaneous recordings. Optical signals were acquired with a long-pass 715 nm filter, a spatial resolution of 700 μm/pixel, and a frame rate of 1 kHz. Wedges were paced by 5 ms pulses on the endocardial or epicardial surfaces, mimicking ectopic activity, or by bipolar stimulation of a free-running PF, mimicking normal anterograde activation through at least one PMJ (see Supplementary Figure S1). Optical signals were measured on each tissue surface and for each pacing configuration, before and after (7 min) the onset of no-flow ischemia.

### Data Analysis

Optical AP signals were filtered using spatial averaging (kernel 2.1 mm) and temporal averaging (kernel 3 ms). Optical ATs were determined by the time of maximal derivative during the AP upstroke and APD was determined as the difference between the time of 90% of repolarization and AT. APDs were measured from a small area (2.8 mm × 2.8 mm) at the origin of activation or the origin of breakthrough, on each surface and depending on the pacing location. For PF pacing, local endocardial APD was measured in a region distinct from all potential origins of activation mediated by PF. Dispersion of APD (μ) was measured as the difference between 5 and 95% confidence interval of normal sample distribution. Epicardial conduction velocities were analyzed using a published MATLAB program (Doshi et al., 2015). Global dispersion of repolarization time (RT) was quantified as the difference between maximal and minimal RT over the whole field of view. Local RT dispersion was also measured in small areas (2.8 mm × 2.8 mm) at the PMJ sites or other endocardial sites.

### Selection of Endocardial PMJs

**FIGURE 1:**
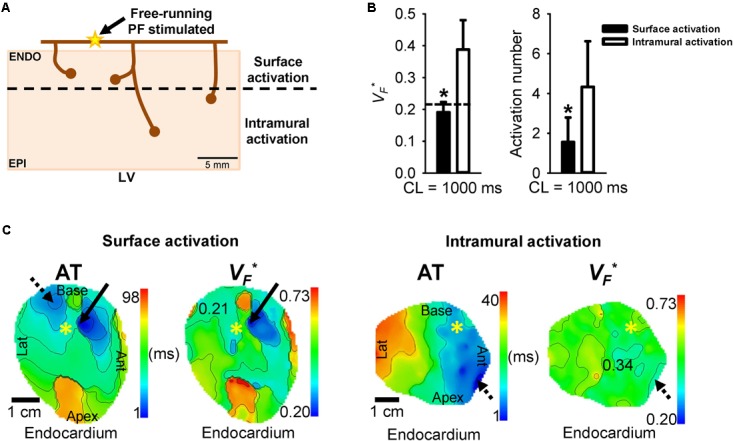
Surface versus intramural PMJ activation determined by *V_F_^∗^* calculation. **(A)** Schematic representation of the origins of myocardial activation from the Purkinje network in sheep (yellow star). Purkinje mediated-activation showed two different transmural activation profiles, a surface activation or an intramural activation. **(B)** Mean ± SD of *V_F_^∗^* or activation number for surface or intramural activation during PF pacing at 1 Hz. Transmural AT was defined as the difference between time at the origin of activation and time at the origin of breakthrough. Dashed line shows *V_F_^∗^* threshold differentiating origins residing at the tissue surface or deeper myocardium. Statistical differences were determined by paired *t*-test (^∗^*p* < 0.05). **(C)** Corresponding endocardial AT and *V_F_^∗^* maps for a short or a long transmural AT during PF stimulation at 1 Hz, by site marked (^∗^). Arrows show origin of early activation and dashed arrows origin of breakthrough. Isochrones are 10 ms spacing.

It is known that the Purkinje network organization can vary across species (Canale et al., 1986). In human hearts, PFs are located subendocardially whereas in ungulates like sheep, a transmural distribution of PFs is found (Shimada et al., 2004; Ono et al., 2009; Yoshimura et al., 2014), giving rise to two origins of activation, superficial layers and intramural layers (**Figure 1A**). To select the location of PMJs at the endocardial surface, and thus focus on activation sequences similar to man, the orientation of the wave front at the origins, with respect to the endocardial surface, was calculated using the normalized amplitude of the maximal derivative of AP upstrokes (*V_F_^∗^*), as previously described (Hyatt et al., 2008; Walton et al., 2012). Here, a low *V_F_^∗^* (<0.22) corresponds to a surface activation and a high *V_F_^∗^* (>0.22) to an intramural activation (Walton et al., 2014).

### Ionic Models

Membrane electrical activity (*I*_m_) for all cell types was based on the Ten Tusscher 2 cell model (ten Tusscher et al., 2004). **Table 1** lists modifications of each model parameter. Modifications were based upon a previous study (Walton et al., 2014) but calcium and sodium current conductances were modified to obtain similar APD values of experimental findings (**Table 1**). Each cell type was subjected to 100 s of pacing at a constant basic CL of 1000 ms for stabilization and the last AP used for comparison of characteristics. States of ionic variables were captured 1 ms prior to stimulation of the last AP.

**Table 1:** Parameter modifiers for ionic models. Modifications were based upon a previous study (Walton et al., 2014) but GCa and GNa currents were modified to obtain similar APD values of experimental findings.

	Control			Ischemia	
Flags	Purkinje	ENDO	EPI	ENDO	EPI
GNa	×2.94			×0.8	×0.8
GCaL				×0.8	×0.8
D_CaL_off	-5.0				
Gto	×3.0	×0.5		×0.5	
GK1		×0.7		×0.7	
Gks	×0.2	×0.55	×0.9	×0.55	×0.9
Gkr	×0.2	×0.55	×0.9	×0.55	×0.9
Xr2_off	-8.0				
knak	×0.2				
knaca	×0.3				
GpCa	×11.0			×0.2	×0.2
GpK	×6.0			×0.5	×0.5
Vrel	×0.6				
Vmaxup	×0.6				
GbCa	×0.2				
Ko	5.4	5.4	5.4	8.0	8.0
F_ATP	0.0	0.0	0.0	×0.08	×0.08

*D_CaL_off, voltage-shift of inactivation of D-type Ca^*2*+^ current; F_ATP, binding kinetics of ATP to the potassium ATPase pump; GbCa, conductance of background Ca^*2*+^; GCaL, L-type Ca^*2*+^ conductance; GNa, Na^+^ current conductance; Gkr, rapid K^+^ conductance; Gks, slow K^+^ conductance; GK1, inward rectifier K^+^ conductance; GpCa, flux of the Ca^*2*+^ pump; GpK, conductance of the K^+^ pump; Gto, transient outward conductance; knaca, flux of the Na^+^–C^*a2*+^ exchanger; knak, flux of Na^+^-K^+^ exchanger; Ko, extracellular potassium ion concentration; Vmaxup, flux of Ca^*2*+^ uptake in to the SR; Vrel, flux of triggered Ca^*2*+^ release from the sarcoplasmic reticulum (SR) compartment; xr2_off, voltage-shift of inactivation of rapid K^+^ current*.

### Human Transmural Ventricular Wedge Model

We used the well-validated Purkinje-myocyte model developed by our group (Deo et al., 2009; Boyle et al., 2013; Zamiri et al., 2014). A finite element model of a three-dimensional transmural ventricular wedge, measuring 5 mm × 5 × 10 mm, was considered at a spatial resolution of 0.1 mm. The model consisted of an epicardial layer (2 mm), while the remaining 8 mm were endocardial. A single PF of length 3 mm was inserted such that fibers terminated at PMJs of depths of either 0.6 or 5 mm from the endocardium. The terminal point of the Purkinje strand is connected to myocardial nodes within a set radius of 600 μm. One cut-transmural face was considered as the imaged face from which the PF was positioned. Electrical activity was solved with the CARP simulator (Vigmond et al., 2003) using the monodomain approach with baseline conductivity value of 0.33 S/m in the longitudinal (σ_L_), transverse (σ_T_), and transmural (σ_S_) directions, corresponding to a conductivity anisotropy ratio of 9:3:1. A 120° linear transmural fiber rotation was implemented. In non-ischemic and ischemic models, we identified the highest Purkinje-myocyte coupling resistance, *R*_PMJ_, to the nearest 42 MΩ, that permitted anterograde transmission, which were 40.4 and 43.0 GΩ, respectively. Anterograde propagation delays were on the order of 7–8 ms. To reproduce previous experimental protocols, the endocardial and the epicardial surfaces were stimulated by intracellular current injection, mimicking ectopic activity. Direct Purkinje stimulation mimicking normal activation (PF to myocardium) was also performed. ATs were determined by a fixed threshold level of -10 mV to avoid capturing artifacts of stimulation or early depolarization of the AP foot manifest by large voltage gradients across the PMJ. APD was measured between activation and a 90% level of repolarization.

### Statistical Analysis

Action potential duration differences from endocardial or epicardial groups were determined by paired *t*-tests and one-way ANOVA for Purkinje-stimulated groups. Statistical differences for transmural gradients were determined by one-way ANOVA and for global dispersion by two-way ANOVA. Comparisons between groups used paired *t*-tests.

## Results

### Determining PMJ Depth

The depths of PMJs were determined by examining the orientation of myocardial excitation centers, relative to the endocardial surface plane, during PF pacing. Origins of activation sites were examined for each experiment (*N* = 7) to differentiate PMJs with surface activation from intramural activation. Of these, 14 PMJs were identified with *V_F_^∗^* values less than a 0.22 threshold (Walton et al., 2014) and 39 were greater, corresponding to surface and intramural origins, respectively (**Figure 1B**). **Figure 1C** shows endocardial AT and *V_F_^∗^* maps for surface and intramural activation origins when pacing PF at 1 Hz. Origins of early activation are indicated by solid arrows and breakthrough sites by dashed arrows.

### APD Distribution and Repolarization Heterogeneity as a Function of Activation Sequence

**FIGURE 2:**
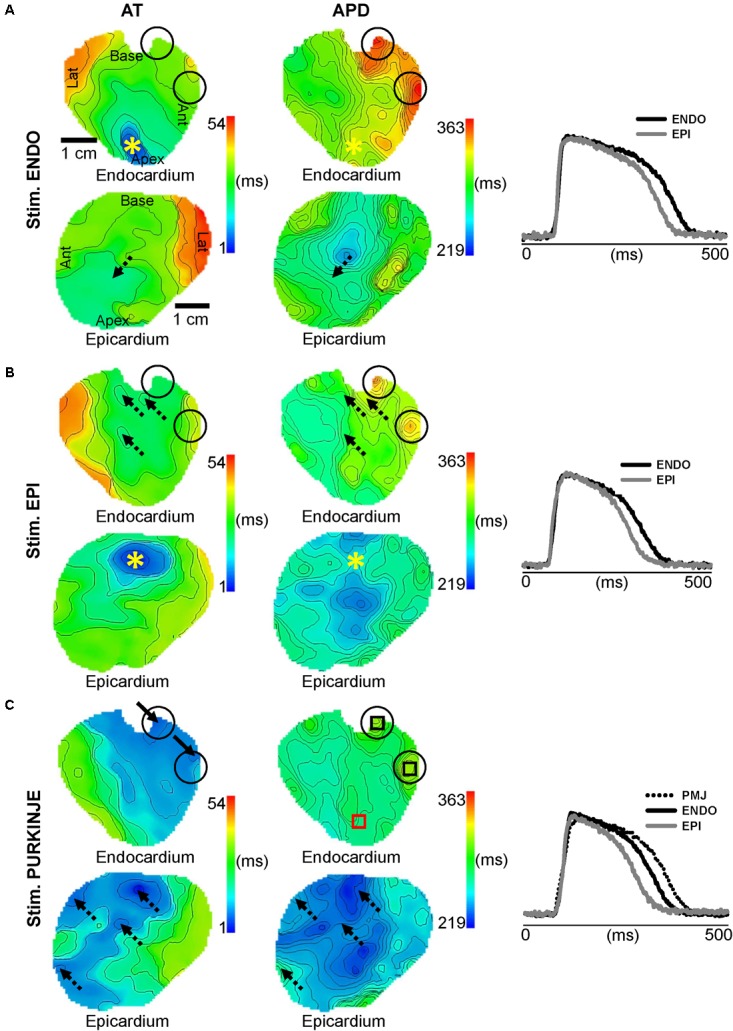
Influence of pacing location on endocardial and epicardial APD. Endocardial and epicardial AT and APD maps and corresponding AP traces derived from LV preparations when pacing at 1 Hz on the ENDO **(A)**, EPI **(B)**, or PF **(C)**, by site marked (^∗^). **(C)** Origins of early activation during PF pacing are localized by arrows. Regions for APD measurements are indicated by the black boxes for PMJs and the red box for ENDO. Dashed arrows show origins of breakthrough. Black circles show PMJs location on AT and APS maps for all pacing sites. Isochrones are 5 ms spacing for AT and APD maps.

Optical images from the endocardial and epicardial surfaces of the sheep were obtained to investigate the relationship between the activation sequence and the transmural distribution of APD_90_. **Figure 2** shows endocardial and epicardial AT and APD_90_ maps and corresponding AP traces derived from LV wedge preparations when pacing the endocardium (ENDO), epicardium (EPI), or free-running PF at 1 Hz. A total AT of 54, 49, and 42 ms were found across the LV when pacing ENDO, EPI, and PF, respectively (left panel). Optical APs from the origin of activation or the origin of breakthrough (dashed arrows), depending on the pacing location, were compared and aligned by the maximal derivative of the upstroke in order to highlight APD differences (right panel). A transmural APD gradient was observed for all pacing locations. During PF stimulation (**Figure 2C**), the spatial distribution of APD_90_ was heterogeneous (μ = 219 ± 84 ms). As previously described (Walton et al., 2014), we observed the longest APD_90_ (315 ms) at sites of earliest endocardial AT compared to later activated regions on ENDO. Although a heterogeneous pattern of APD_90_ was also found during ENDO (μ = 213 ± 100 ms) and EPI (μ = 231 ± 109 ms) pacing, the longest APD_90_ was not found at the sites of stimulation but at the endocardial PMJs. Finally, dispersion of repolarization was not significantly different between ENDO and PF pacing, but was increased when pacing the epicardium (see below).

### Modulation of the Transmural APD Heterogeneity by the Activation Sequence

**FIGURE 3:**
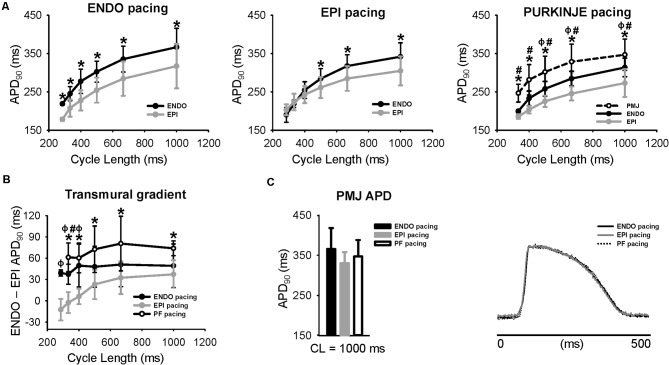
Dependence of transmural APD heterogeneity on the activation sequence. **(A)** Frequency-dependence of transmural differences of APD for ENDO (*n* = 7), EPI (*n* = 7), and PF (*n* = 7) stimulation sites. APD measurements were performed at origins of activation or origins of breakthrough on each surface for each stimulus location (black box shown in **Figure 2**). **(B)** Transmural differences of APD when pacing ENDO, EPI, and PF at different frequencies. **(C)** Mean ± SD APD at the PMJ for ENDO, EPI, or PF stimulation at 1 Hz (upper panel) and corresponding AP traces (lower panel). CL, cycle length. Statistical differences were determined by paired *t*-tests for ENDO and EPI pacing (^∗^*p* < 0.05), one-way repeated measures ANOVA for PF stimulation (PMJ vs EPI, ^∗^*p* < 0.05; PMJ vs ENDO, #*p* < 0.05; ENDO vs EPI, aaa*p* < 0.05), and one-way ANOVA for transmural gradient (PF vs EPI pacing, ^∗^*p* < 0.05; PF vs ENDO pacing, #*p* < 0.05; ENDO vs EPI pacing, aaa*p* < 0.05).

**Figure 3A** shows dynamics APD restitution from ENDO, EPI and PF stimulations, ranging from 286 (effective refractory period) to 1000 ms for ENDO and EPI pacing and from 333 (effective refractory period) to 1000 ms for PF pacing. APDs were measured locally at origin of activation or origin of breakthrough on the opposite surface, as shown in **Figure 2**. **Figure 3B** indicates transmural APD gradients between ENDO and EPI (endocardial–epicardial APDs), when pacing ENDO, EPI, or PF at different pacing CL. We observed a significant transmural gradient for PF pacing for all frequencies tested, with PMJ APD = 347 ± 41 ms and EPI APD = 273 ± 36 ms (gradient = 74 ± 10 ms, *p* < 0.001). A similar transmural gradient was observed, albeit reduced, when pacing ENDO (49 ± 31 ms; *p* = 0.005). EPI pacing revealed the smallest transmural APD gradient (37 ± 20 ms; *p* = 0.003) and for all pacing frequencies, EPI gradient was significantly lower from Purkinje gradient (*p* = 0.018). At higher frequencies, EPI APD (224 ± 22 ms) became greater than ENDO APD (219 ± 10 ms) when pacing EPI and transmural gradient became negative (-2 ± 15 ms) and significantly different to ENDO (37 ± 14 ms, *p* = 0.003). **Figure 3C** shows PMJ APD and corresponding AP traces when pacing ENDO, EPI, and PF; respectively at 1 Hz. Interestingly, there was no significant APD difference (*p* > 0.05), and the longest APD remains at PMJ site independently of the pacing location, as shown in **Figure 2**.

### Modulation of APD Dispersion During Acute Ischemia

**FIGURE 4:**
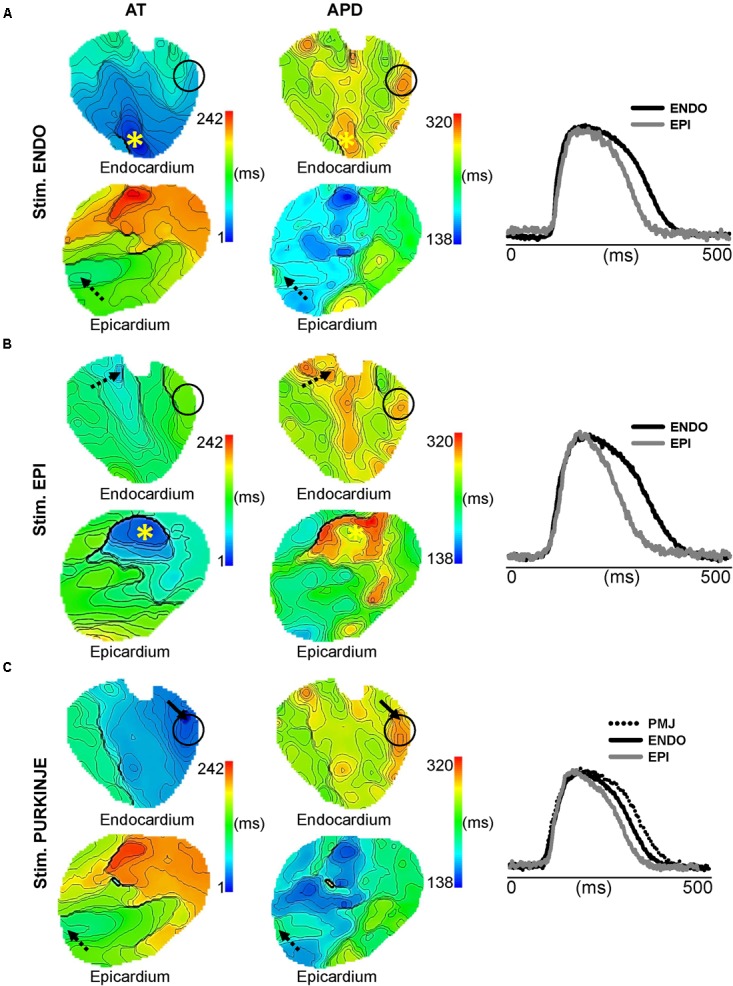
Impact of ischemia on transmural APD heterogeneity. Endocardial and epicardial AT and APD maps and corresponding AP traces as shown in **Figure 2** for ENDO **(A)**, EPI **(B)**, and PF **(C)** pacing. Stimulation sites are indicated by (^∗^), arrows show origins of early activation for PF stimulation in **C**, dashed arrows indicate origin of breakthrough, and black circles show PMJs location on AT and APD maps for all pacing sites. Isochrones are 5 ms spacing for endocardial maps and 10 ms for epicardial maps.

During acute ischemia, we observed a loss of anisotropic propagation, a shortening of APD, a significant decrease in longitudinal (68 ± 10 vs. 55 ± 7 cm/s) and transverse (25 ± 7 vs. 13 ± 4 cm/s) conduction velocities, and a significant increase of their ratio (*p* < 0.05; see Supplementary Figure S2), as previously described (Kleber et al., 1987; Mizumaki et al., 1993). We also observed an increase in post-repolarization refractoriness in myocardium by 84 ms, but not at the PMJs (not shown). **Figure 4** shows endocardial and epicardial AT and APD_90_ maps and corresponding AP traces, when pacing ENDO, EPI, and PF at 1 Hz, as in **Figure 2**, but after 7 min of no-flow ischemia. A prolongation of total AT across the LV was found for all stimulation sites (242 ms ENDO; 191 ms EPI, and 242 ms PF) compared to control conditions (**Figure 2**, left panel). The corresponding transmural gradient of APD_90_ was more pronounced for all pacing locations (156 ms ENDO; 145 ms EPI; 154 ms PF) compared to control (**Figure 2**, middle panel). However, the pacing location had no bearing on the transmural APD gradient (right panel). Interestingly, for all pacing sites, the spatial distribution of APD_90_ (μ = 227 ± 106 ms ENDO; μ = 223 ± 78 ms EPI; μ = 254 ± 80 ms PF) was heterogeneous. Furthermore, the APD_90_ at PMJ sites was preserved during ischemia, although loss of junctions was observed in some cases when compared to control (see **Figure 2C**).

### Influence of Ischemia on the Transmural APD Gradient and Repolarization Dispersion

**FIGURE 5:**
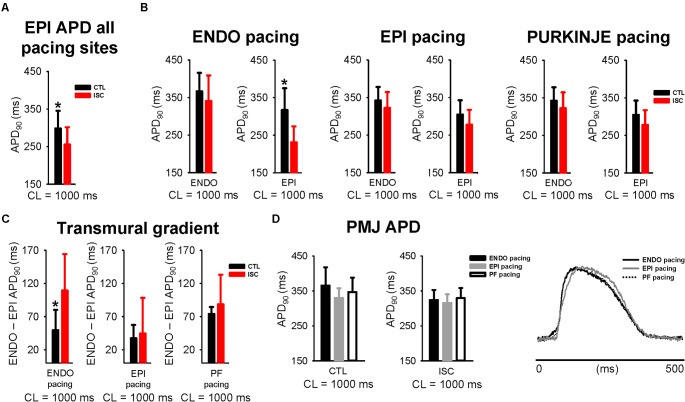
Greater resistance of Purkinje network to ischemia. **(A)** Mean ± SD EPI APD across all pacing locations at 1 Hz, before and after 7 min of ischemia. CTL, control; ISC, ischemia. **(B)** Mean ± SD APD from ENDO and EPI surfaces for each stimulus location stimulation at 1 Hz (before and after ISC). **(C)** Transmural differences of APD when pacing ENDO, EPI, and PF at 1 Hz, before and after ISC. **(D)** Mean ± SD PMJ APD (upper panel) and corresponding AP traces (lower panel), as in **Figure 3**, are shown for all pacing locations, before and after ISC. Statistical differences were determined by paired *t*-tests (^∗^*p* < 0.05).

**FIGURE 6:**
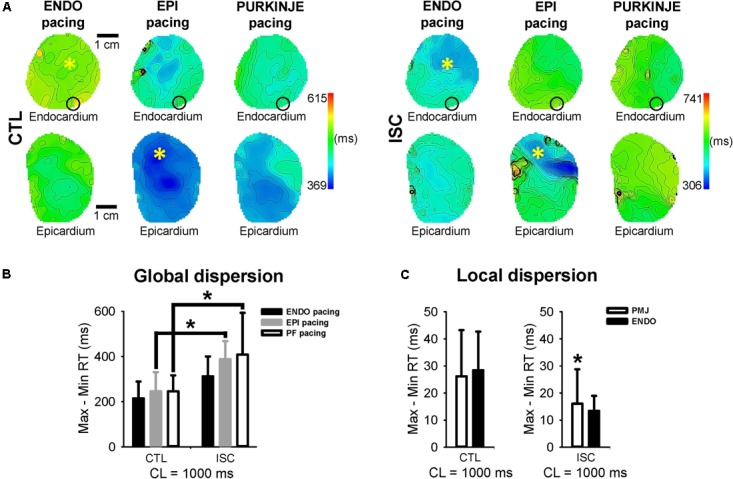
Impact of pacing sequence on dispersion of repolarization during acute ischemia. **(A)** RT endocardial and epicardial maps for the different pacing modalities in control conditions and after 7 min of ischemia. Stimulation sites are indicated by (^∗^) and black circle shows PMJ location for all pacing sites. Isochrones are 10 ms spacing. CTL, control; ISC, ischemia. **(B)** Mean ± SD global RT dispersion when pacing ENDO, EPI, and PF at 1 Hz, before and after ISC. Statistical differences were determined by two-way ANOVA (^∗^*p* < 0.05). **(C)** Mean ± SD local RT dispersion at PMJs compared to endocardium, before and after ISC. Statistical differences were determined by paired *t*-test (^∗^*p* < 0.05).

We found that the mean ENDO APD across all pacing locations tended to decrease after 7 min of ischemia from 352 ± 42 to 336 ± 47 ms (*p* = 0.188; data not shown) and the mean EPI APD was significantly reduced from 298 ± 47 to 255 ± 46 ms (*p* = 0.005; **Figure 5A**). **Figure 5B** shows comparisons of APD from endocardial and epicardial surfaces during ENDO, EPI, and PF stimulation at 1 Hz, before (CTL) and after (ISC) ischemia. APDs were measured locally as shown in **Figure 2**. **Figure 5C** indicates transmural APD gradient depending on the pacing location at 1 Hz, in control or ischemic conditions. The observed degree of APD reduction was strongly dependent on the activation sequence, with significant APD reduction on the epicardium when pacing ENDO, but not when pacing EPI (**Figure 5B**). This has an impact on the transmural APD gradient, which was significantly increased when pacing ENDO compared to control (109 ± 55 ms, *p* < 0.050; **Figure 5C**). No significant changes in transmural gradients were observed for PF pacing in control versus ischemic conditions (*p* = 0.42). Interestingly, the PMJs for which conduction was lost during ischemia also failed to exhibit prolonged APDs during ischemia (see the black circle in **Figure 4C** and compare with **Figure 2C**). **Figure 5D** shows PMJ APD and corresponding AP traces, as in **Figure 3**, for all pacing locations, before and after ischemia. As in control conditions, PMJ APD did not depend on pacing location (*p* > 0.05); the longest APD remained at the PMJ site. PMJ APD was resistant to ischemia, as shown in **Figure 4**. **Figure 6A** shows endocardial and epicardial RT maps for the three pacing locations in control and ischemic conditions. In control conditions, activation sequence had no effect on global RT dispersion (**Figure 6B**). However, during ischemia, there was a trend for increased global RT dispersion, which was significant for EPI and PF pacing (note the different scale bars). Interestingly, in control conditions, there was no significantly increased RT dispersion during PF pacing at the PMJ sites when compared to ENDO, but it was the case during ischemia (**Figure 6C**).

### Ionic Models

**FIGURE 7:**
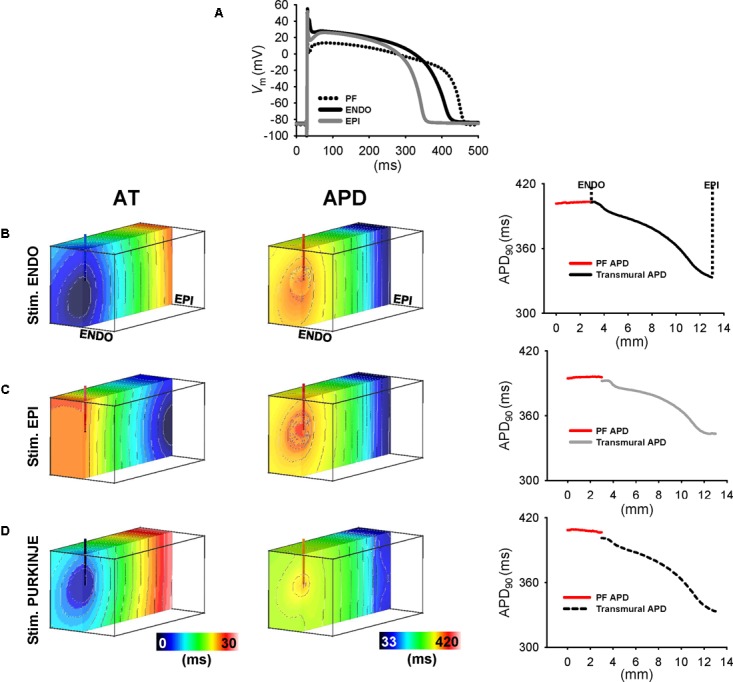
Transmural APD heterogeneity in human ventricular wedge model. **(A)** APs derives from human from PF, endocardial, and epicardial ionic models of control heart. Shown are the last of a train of 100 APs stimulated at 1 Hz. AT and APD maps in simulations from transmural cross-sections of wedges for ENDO **(B)**, EPI **(C)**, and PF **(D)** stimulation at 1 Hz. Right panels show transmural APD profiles intersecting the PMJ and APD profiles along the free-running PF (red line). Isochrones are 2 ms spacing for AT maps and 5 ms for APD maps.

**FIGURE 8:**
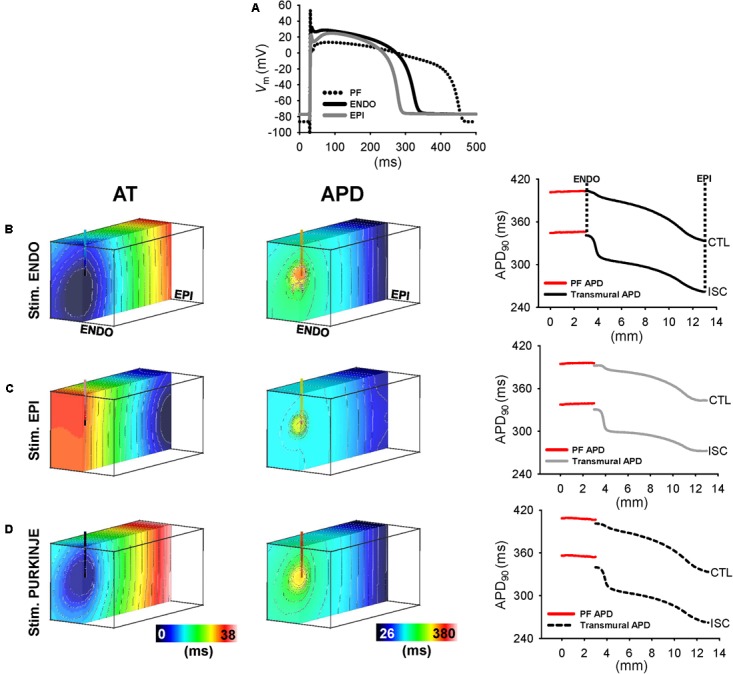
Transmural APD heterogeneity in ischemic human model. **(A)** APs derived from human from PF, endocardial, and epicardial ionic models of ischemic heart. The last of a train of 100 APs are shown. Same PF control AP was used in ischemic condition due to Purkinje resistance to ischemia. AT and APD maps and APD profiles are shown for simulations of ischemia following ENDO **(B)**, EPI **(C)**, and PF **(D)**.

**Figures 7A**, **8A** show that the Purkinje cell was electrophysiologically distinct from control and ischemic working cardiomyocytes. In control myocardial cells, the Purkinje cell exhibited a more pronounced AP spike (47.3 mV) and a more negative plateau (13.4 mV) compared to ENDO (44.2 and 28.0 mV, respectively) and EPI cells (40.6 and 26.1 mV, respectively). The same Purkinje ionic model was used in control and ischemic conditions due to Purkinje resistance to ischemia (Henry and Lowry, 1983; Ono et al., 2009). Ischemia was modeled according to previously published methods (Kazbanov et al., 2014) and APD was modified for consistency with experiments using ionic modifies shown in **Table 1**. The resulting AP spike and plateau amplitudes for ischemic myocardial cells with ENDO were 32.4 and 29.0 mV, respectively, and were 32.2 and 24.1 mV, respectively, for EPI cells. In control conditions, APDs of ENDO, EPI, and PF cells were 434, 358, and 466 ms, and APDs were reduced to 346 and 296 ms in ischemia for ENDO and EPI cells.

### Modulation of the Transmural APD Heterogeneity Validated by Human Wedge Simulations

Simulations were performed at 1 Hz pacing from ENDO, EPI, and PF to provide, further than in experiments, continuous transmural profiles of activation and repolarization, in addition to their properties local to and across the PMJ. AT and APD maps of transmural cross-section intersecting the PMJ and corresponding APD profiles were derived for control (**Figure 7**) and ischemic (**Figure 8**) myocardium. In general, APD negatively correlated with AT. Yet prolongation of APD was always observed at the PMJ and corresponded to the maximum observable APD in all simulations. In control simulations (**Figure 7**), ENDO and PF pacing developed APD gradients of 70 and 68 ms, respectively, whereas transmural APD differences were only 49 ms for EPI stimulation. APD at the PMJ was greatest in myocardial nodes when excitation was elicited from PF to the myocardium (407 ms) versus 403 and 396 ms for ENDO and EPI origins, respectively. Ischemic myocardium (**Figure 8**) showed global shortening of APD versus controls (295 ± 20 vs. 373 ± 21 ms; data are PF pacing). An increasing APD gradient from EPI to ENDO was preserved but increased for all pacing locations: ENDO (79 ms); EPI (58 ms); and PF (78 ms), compared with controls. Comparison between local neighborhood APD at 1.5 mm from PMJ and PMJ APD in myocardium showed APD elevation at the PMJ for ENDO (10 ms), EPI (7 ms), and PF (8 ms) stimulations. This effect was more pronounced during ischemia and for all pacing sites (33 ms ENDO; 31 ms EPI; 31 ms PF). Moreover, free-running PF APD in control and ischemic (408 ± 1 and 356 ± 1 ms; data are PF pacing) conditions revealed the presence of a PF-myocardium gradient which was more elevated during ischemia (61 ms) compared to control (35 ms). Thus, APD modulation at the PMJ was more pronounced in ischemia versus control.

### Modulation of Sheep Functional PMJs by Ischemia

**FIGURE 9:**
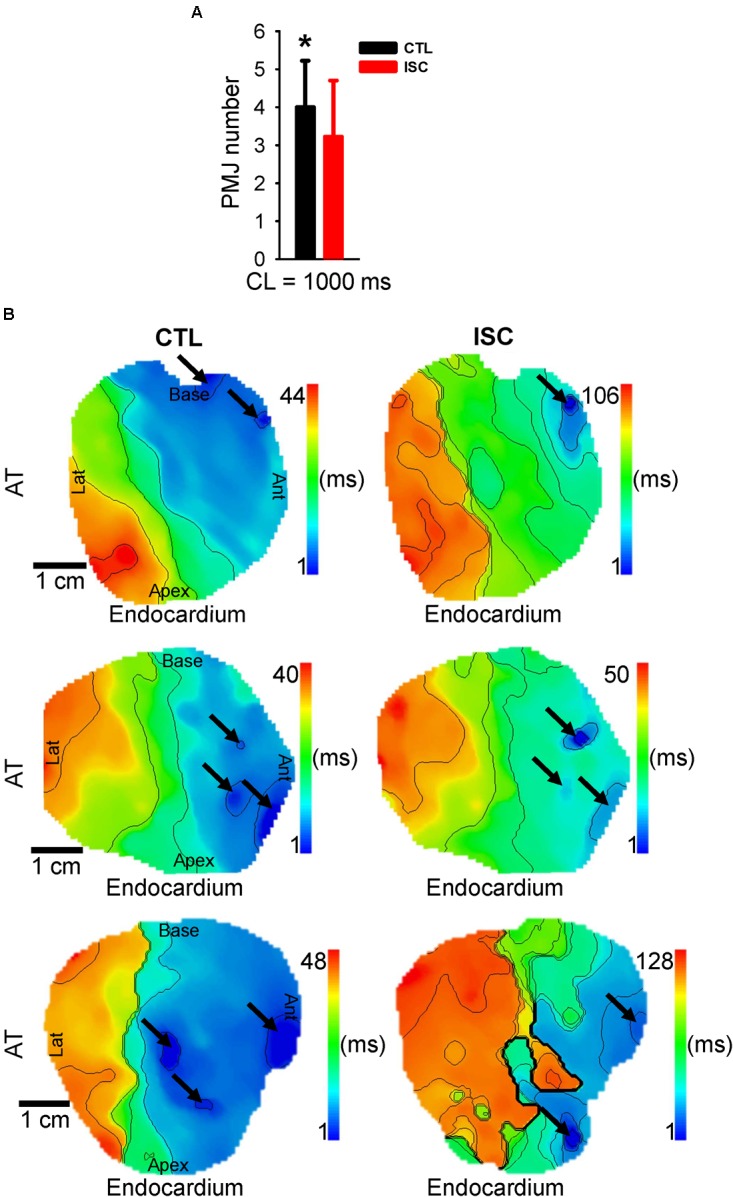
Quantification of sheep functional PMJs before and after ischemia. **(A)** Mean ± SD number of activation origins when pacing PF at 1 Hz. **(B)** Corresponding endocardial AT maps for three different wedge preparations. Arrows show origins of early activation corresponding to PMJs. Isochrones are 10 ms spacing. Statistical differences were determined by paired *t*-test (^∗^*p* < 0.05).

**Figure 9A** indicates the number of all activation origins (surface and intramural) when pacing PF at 1 Hz before control (CTL) and after ischemia (ISC). A significant decrease of PMJ number was observed in ischemic conditions compared to control (*p* = 0.031). **Figure 9B** shows endocardial AT maps for three different wedge preparations illustrating three different modulations of functional PMJs during ischemia, loss of a functional junction (top panel), identical functional junctions (middle panel), or new functional junction (bottom panel). As described above, a loss of function of a PMJ during ischemia was also evident from the APD maps.

## Discussion

This present study aimed to characterize the influence of the activation sequence on dispersion of ventricular repolarization in the healthy and ischemic myocardium. Such mechanisms were investigated experimentally and computationally by comparing sheep LV before and after the onset of no-flow ischemia. Recently, it was shown that in addition to myocardial heterogeneity, sites of functional PMJs contribute to APD heterogeneity in healthy myocardium. Using optical imaging, we found in control conditions that EPI stimulation reduces the transmural APD gradient compared with PF and ENDO stimulation (**Figures 2**, **3**), but has no effect on global RT dispersion as ENDO and PF pacing sites (**Figure 6**). In ischemic conditions, the transmural gradient following PF pacing was preserved and local dispersion of RT was elevated at the PMJ sites (**Figures 4**–**6**). The results were consistent with simulations (**Figures 7**, **8**).

### Electrotonic Modulation of APD Heterogeneity

It has been previously demonstrated that activation sequence influences epicardial APD when the propagation of activation is transverse to myocardial fiber orientation (Osaka et al., 1987; Gotoh et al., 1997). In terms of transmural APD, previous studies limited to human and rabbit (Glukhov et al., 2010; Myles et al., 2010) LV cut surfaces showed that electrotonic influences are important in determining the transmural repolarization sequence in ventricular myocardium and that they are sufficient to overcome intrinsic differences in the electrophysiological properties of the cells across the ventricular wall (Myles et al., 2010). In the current study, optical measurements from endocardial and epicardial surfaces of sheep LV revealed APD characteristics comparable to those recorded from the cut surface. We demonstrated the presence of increased endocardial APD when compared to the epicardium. However, the magnitude of the gradient was dependent upon pacing location, with the transmural APD gradient strongly reduced during EPI stimulation. However, we found that endocardial and epicardial spatial distributions of APD were not modulated by the activation sequence (**Figure 2**), most likely owing to the AP morphology and repolarization ionic currents, which are similar to pig as shown previously (Walton et al., 2013).

### Role of Purkinje Fibers on Repolarization Patterns

This study has confirmed previous results obtained by our team showing that PF coupled to the myocardium modulates locally the APD in the myocardium (Walton et al., 2014). We found during PF pacing the longest APD at sites of earliest endocardial AT compared to later activated regions on ENDO (**Figures 2**, **3**). This corresponds to the role of PF in modulating heterogeneity of repolarization as shown previously (Walton et al., 2014). Unexpectedly, during ENDO and EPI stimulations, longest APDs were found at similar sites as the PMJ (**Figure 2**) and there was no significant PMJ APD difference depending on the pacing location (**Figure 3**). Results show that PMJ APDs are not modulated by the activation sequence or acute ischemia, but mainly depend upon coupling of PFs with myocardium. Moreover, it is well known that PFs in sheep, unlike human, have transmural distribution, which enables both surface and intramural origins of activation. These were determined from the normalized amplitude of the maximal derivative of AP upstrokes (*V_F_^∗^*) (Hyatt et al., 2008; Walton et al., 2012) and correlate to APD heterogeneity (Walton et al., 2014). Within the population of sheep LV preparations, we observed two types of transmural activation profiles when stimulating through PFs: ENDO to EPI activation and simultaneous EPI and ENDO activation (**Figure 1**). Even with such PF architecture, transmural APD gradients and repolarization heterogeneity were similar between ENDO and PF pacing. Our study is the first to investigate the role of the Purkinje network in the modulation of repolarization gradients in the healthy myocardium of large mammals.

### Acute Ischemia and Dispersion of Repolarization

We used a model of acute ischemia corresponding to 7 min no-flow in coronary-perfused tissue that showed a diminution of APD, a significant decrease in longitudinal and transversal conduction velocities, and an increase of conduction anisotropy (see Supplementary Figure S2), as previously described (Kleber et al., 1987; Mizumaki et al., 1993; Cascio et al., 1995). Acute ischemia in our experimental preparations (**Figure 4**) was associated with a prolongation of total AT for all pacing locations compared to control conditions (**Figure 2**). Transmural APD gradients were greater during acute ischemia compared to control. This may be partially explained by the differential ATP sensitivity of ATP-regulated K’ channels across the ventricular wall which are more sensitive in EPI (Shaw and Rudy, 1997; Boyle et al., 2013) and therefore more susceptible to the decrease of ATP during ischemia (Shaw and Rudy, 1997; Scheinman, 2009). APD maxima remained localized to PMJs, in ischemic (**Figure 5**) conditions with little change in APD compared to control. The preservation of PFs and their electrophysiological state during ischemic insult has previously been documented (Tanaka et al., 2005; Ono et al., 2009). This phenomenon is thought to occur due to high glycogen levels permitting resistance to hypoxia. Interestingly, we found that during ischemia, coupling at certain PMJs could be lost. This manifested as local conduction failure in to the myocardium as well as the lack of local APD maxima. Future studies will be required to understand why some PMJs lose their function during ischemia. In terms of repolarization heterogeneity, we found that global dispersion of RT was increased for all pacing modes in ischemia. However, at a local level, APD and RT gradients are significantly increased during ischemia in the vicinity of functional PMJs.

### Clinical Implications

T wave morphology is associated with risk of sudden cardiac death (Zhang et al., 2000). Transmural repolarization heterogeneity is believed to underlie the T wave in humans, although recent evidence points to a more prominent role for basico-apical and left-right differences (Boukens et al., 2015, 2016). The end of the T wave is governed by the latest repolarizing zone, typically associated to cells with the longest APD, previously thought to be the so-called M cells (Sicouri and Antzelevitch, 1991; Glukhov et al., 2010). But according to this study, PFs appear to have an important role in the dispersion of repolarization, and even more so during acute ischemic insult. Earlier studies using a canine VF infarction model showed that PFs survived acute ischemia and were found to be involved in both triggered and reentrant arrhythmias (Tabereaux et al., 2007; Dosdall et al., 2008). The Purkinje system has been shown to be responsible for the initiation of VF in patients with no structural heart disease (Haissaguerre et al., 2002a,b) and in patients with post-infarct ischemia, which appear to be driven by Purkinje-like triggered activity originating from the scar border zone (Marrouche et al., 2004; Szumowski et al., 2004). However, mechanisms underlying these arrhythmias at tissue level are still poorly understood and clinical studies to investigate the influence of the Purkinje system as an arrhythmogenic substrate of VF in healthy or diseased myocardium represent a real challenge owing to the lack of ultrahigh density mapping tools. Our results confirm earlier hypotheses that both PFs and PMJs can remain functional during acute ischemia. Furthermore, we show that for such PMJs, a strong gradient in repolarization exists with the surrounding myocardium. This together with increased post-repolarization refractoriness of the ischemic endocardium could provide an explanation for the post-MI VF vulnerability to short-coupled triggers.

### Limitations

Optical mapping of electrical activity directly from the transmural surface was not performed in this study, as done previously by others (Glukhov et al., 2010), and therefore the full transmural gradient could not be assessed. This approach requires imaging from a cut surface whereby a tissue boundary is artificially imposed on the tissue preparation at the imaged surface. Tissue boundaries have a direct impact on electrical loading conditions and thus amplify apparent repolarization heterogeneities of tissue. Instead, we sought to quantify electrotonic modulation of repolarization and the influence of PF coupling from intact myocardium to preserve *in situ* repolarization heterogeneities. Furthermore, our preparations preserved the structural integrity of the PF network and enabled activation from the PF network via stimulation of free-running PFs. Nevertheless, many parameters are missing to characterize completely the PMJ, especially in human hearts which have a different distribution of the network. It was therefore important to constrain measurements of Purkinje influences on local myocardium to PMJs that lie in the sub-endocardium. This was to enable localization and measurement at the PMJ itself, rather than merely a breakthrough site distant from the PMJ. This was particularly important to identify the true impact on APD (typically prolonged at the PMJ), have a measure of the subsequent delayed RT, and focus on activation sequences that are more relevant to human. Moreover, this current study did not investigate trigger mechanisms of ventricular arrhythmias but provided a potential role of PF as a local and global modulator of repolarization heterogeneity.

## Conclusion

This study demonstrates that PFs play an important role in repolarization dispersion in the sheep LV. Coupling of PFs with myocardium at PMJs results in localized elevation of APD and dispersion of repolarization. Furthermore, PMJs can remain active following ischemia and continue to exert significant influence on local activation and repolarization patterns.

## Author Contributions

MM, RW, and OB conceived and designed research. MM, RW, JB, and EV performed the experiments. MM and RW analyzed the data. MM, RW, and OB interpreted results of the experiments. MM drafted the manuscript. RW, OB, MéH, and MiH edited and revised the manuscript. MM, RW, JB, MiH, EV, MéH, and OB approved the final version of the manuscript.

## Conflict of Interest Statement

The authors declare that the research was conducted in the absence of any commercial or financial relationships that could be construed as a potential conflict of interest.

## Abbreviations

AP: action potential
APD: action potential duration
AT: activation time
CL: cycle length
PF: Purkinje fiber
PMJ: Purkinje-muscle junction
σσ_L_: longitudinal conductivity
σσ_S_: transmural conductivity
σσ_T_: transverse conductivity
VF: ventricular fibrillation.
