# Supplementary material for: Role of the Purkinje-Muscle Junction on the Ventricular Repolarization Heterogeneity in the Healthy and Ischemic Ovine Ventricular Myocardium
Source: Front Physiol. 2018 Jun 14;9:718. doi: 10.3389/fphys.2018.00718 (PMC6010581; doi:10.3389/fphys.2018.00718)
Supplement: Supplementary file 1 [file Presentation_1.pdf]

Supplementary Material

Role of the Purkinje-muscle junction on the ventricular action potential duration heterogeneity in the healthy and ischemic ventricular myocardium

Marine E. Martinez*, Richard D. Walton, Jason D. Bayer, Michel Haïssaguerre, Edward J. Vigmond, Mélèze Hocini and Olivier Bernus

*** Correspondence:**
Corresponding Author: Marine Martinez

L'Institut de Rythmologie et Modélisation Cardiaque (LIRYC)
CRCTB U1045
Hopital Xavier Arnozan,
Avenue du Haut Lévêque,
33604 Pessac,
France.

Tel: +33 (0) 5 35 38 19 54
Fax: +33 (0) 5 35 38 19 98

marine.martinez@ihu-liryc.fr

Supplementary Figures

Supplementary Figure 1

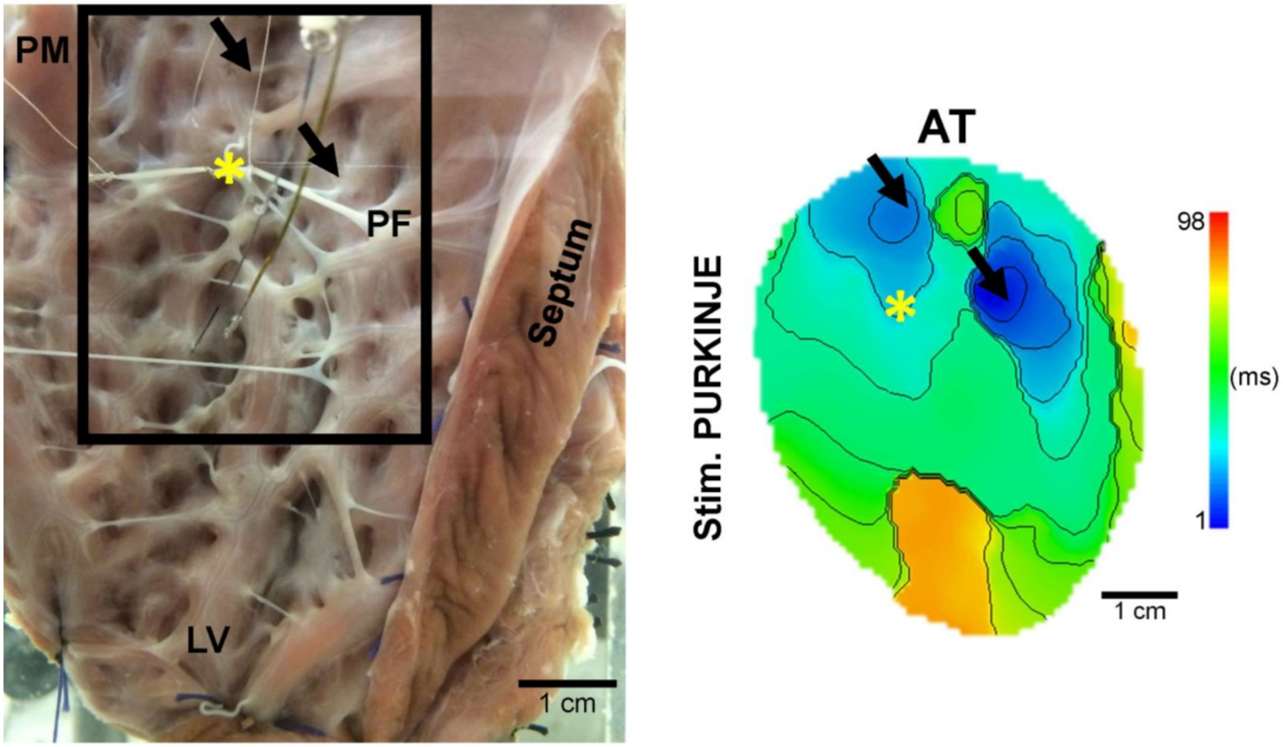

Supplementary Figure 1. Localization of PMJs by free-running PF pacing. Endocardial view of an experimental preparation and corresponding AT map during PF pacing at 1 Hz. Yellow star indicates bipolar free-running PF stimulation and black arrows show activation origins corresponding to PMJs.

Supplementary Figure 2

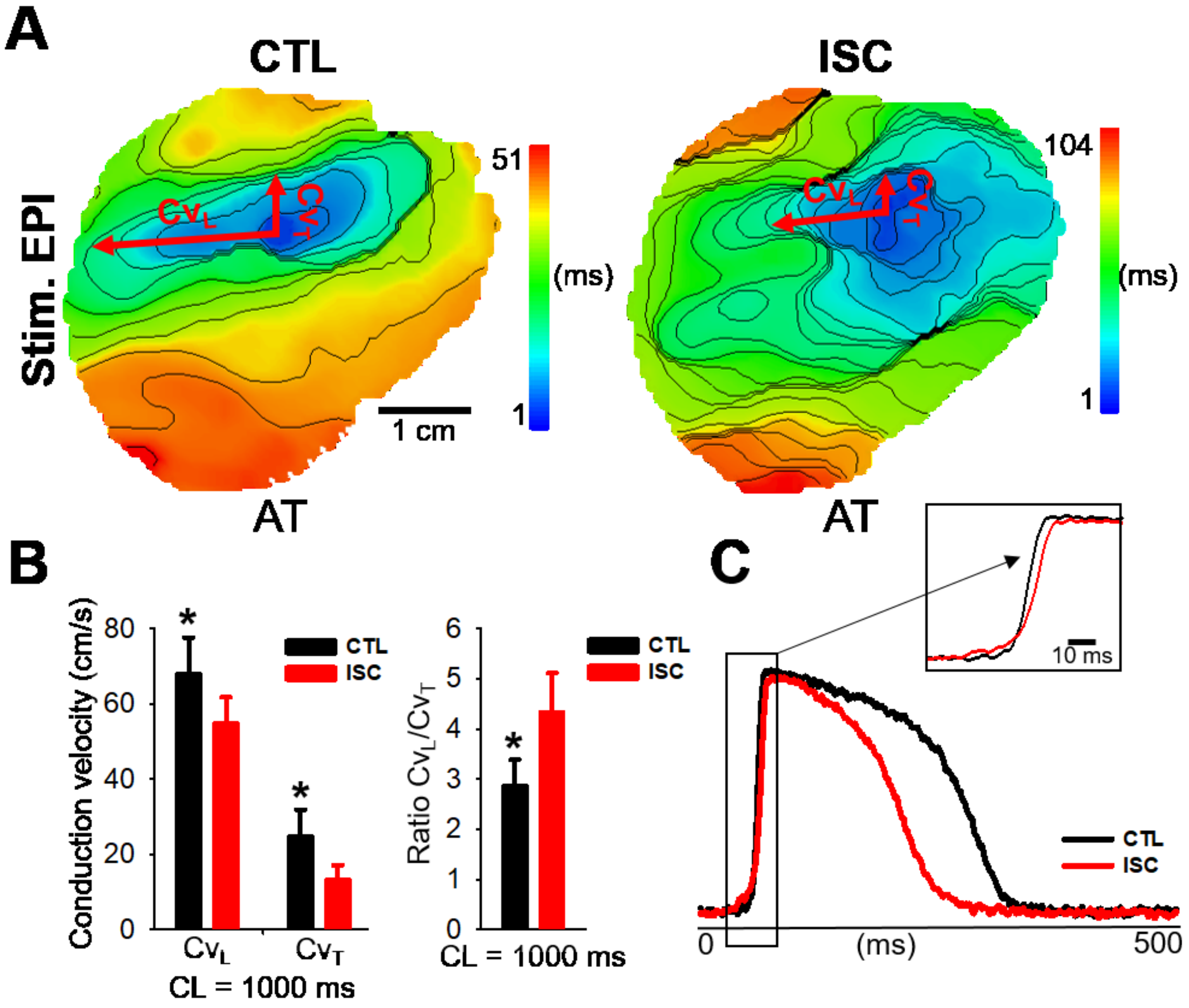

Supplementary Figure 2. Conduction velocity before and after the onset of no-flow ischemia. A, Epicardial AT maps during EPI stimulation at 1 Hz, before and after 7 min of no-flow ischemia. Isochrones are 5 ms spacing. CTL=control, ISC=ischemia. **B**, Longitudinal (CvL) and transversal (CvT) conduction velocity and their ratio from EPI surface when pacing EPI at 1 Hz, in control versus ischemic conditions. **C**, AP traces and corresponding upstrokes extracted from the origin of activation when pacing EPI at 1 Hz, during CTL or ISC. Statistical differences were determined by paired t-tests (*$p<0.05$).
